# Epidemiologic Features of the Monkeypox Outbreak and the Public Health Response — United States, May 17–October 6, 2022

**DOI:** 10.15585/mmwr.mm7145a4

**Published:** 2022-11-11

**Authors:** Christine Marie Kava, Dallas M. Rohraff, Bailey Wallace, Jennifer L. Mendoza-Alonzo, Dustin W. Currie, Anna E. Munsey, Nicole M. Roth, Jonathan Bryant-Genevier, Jordan L. Kennedy, Daniel L. Weller, Athalia Christie, Jennifer H. McQuiston, Peter Hicks, Penelope Strid, Emily Sims, Maria E. Negron, Kashif Iqbal, Sascha Ellington, Dawn K. Smith

**Affiliations:** ^1^CDC Monkeypox Emergency Response Team; ^2^Epidemic Intelligence Service, CDC; ^3^Oak Ridge Institute for Science and Education, Oak Ridge, Tennessee.

On May 17, 2022, the Massachusetts Department of Health announced the first suspected case of monkeypox associated with the global outbreak in a U.S. resident. On May 23, 2022, CDC launched an emergency response (*1*,*2*). CDC’s emergency response focused on surveillance, laboratory testing, medical countermeasures, and education. Medical countermeasures included rollout of a national JYNNEOS vaccination strategy, Food and Drug Administration (FDA) issuance of an emergency use authorization to allow for intradermal administration of JYNNEOS, and use of tecovirimat for patients with, or at risk for, severe monkeypox. During May 17–October 6, 2022, a total of 26,384 probable and confirmed* U.S. monkeypox cases were reported to CDC. Daily case counts peaked during mid-to-late August. Among 25,001 of 25,569 (98%) cases in adults with information on gender identity,^†^ 23,683 (95%) occurred in cisgender men. Among 13,997 cisgender men with information on recent sexual or close intimate contact,^§^ 10,440 (75%) reported male-to-male sexual contact (MMSC) ≤21 days preceding symptom onset. Among 21,211 (80%) cases in persons with information on race and ethnicity,^¶^ 6,879 (32%), 6,628 (31%), and 6,330 (30%) occurred in non-Hispanic Black or African American (Black), Hispanic or Latino (Hispanic), and non-Hispanic White (White) persons, respectively. Among 5,017 (20%) cases in adults with information on HIV infection status, 2,876 (57%) had HIV infection. Prevention efforts, including vaccination, should be prioritized among persons at highest risk within groups most affected by the monkeypox outbreak, including gay, bisexual, and other men who have sex with men (MSM); transgender, nonbinary, and gender-diverse persons; racial and ethnic minority groups; and persons who are immunocompromised, including persons with advanced HIV infection or newly diagnosed HIV infection.

## Epidemiology of Cases

On June 23, 2022, the Council of State and Territorial Epidemiologists approved designating monkeypox as a nationally notifiable disease. Data reported to CDC** by jurisdictions included patient demographic characteristics, history of possible exposure, diagnostic studies performed, and clinical signs and symptoms at onset. Characteristics of monkeypox cases reported during May 17–October 6, 2022, are described and compared before and after July 18, 2022, when five commercial laboratories began testing and substantially expanded capacity and access. This surveillance activity was reviewed by CDC and was conducted consistent with applicable federal law and CDC policy.^††^

**FIGURE 1:**
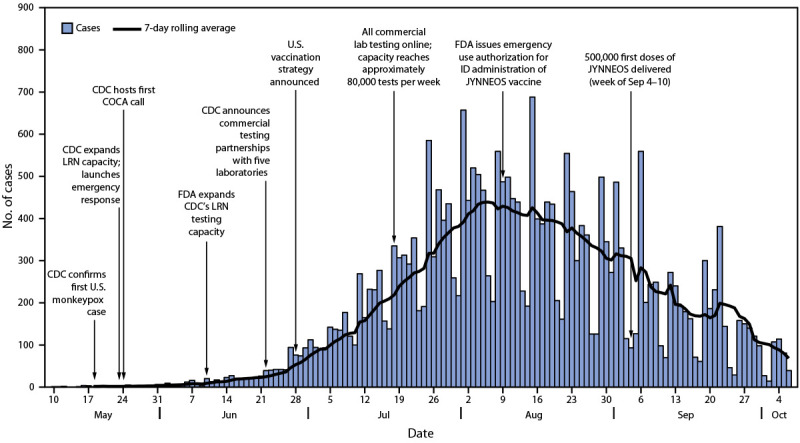
Monkeypox cases* and public health response, by date^†^^,^^§^ — United States, May 17–October 6, 2022 **Abbreviations:** COCA = Clinician Outreach and Communication Activity; FDA = Food and Drug Administration; ID = intradermal; LRN = laboratory and response network. * N = 26,384. Figure excludes one case for which information needed to calculate date is missing. ^†^ Date is defined as the earliest date available among the following: 1) a positive laboratory test report date, 2) CDC call center reporting date, or 3) case data entry date into CDC’s Data Collation and Integration for Public Health Event Responses platform. ^§^ Data since approximately September 25 are incomplete because of delays in reporting.

During May 17–October 6, 2022, a total of 26,384 monkeypox cases were reported to CDC by all 50 states, the District of Columbia, and Puerto Rico. Daily case counts peaked during mid-to-late August (Figure 1). Among 25,678 (97%) persons with monkeypox for whom demographic data were available, the median age was 34 years (range = 0–89 years). From May 17–July 17 to July 18–October 6, the percentage of cases among persons aged 18–29 years increased from 20% to 27%; the percentage of cases among persons aged 30–39 years decreased from 45% to 41% (Table). Among 21,211 (80%) cases in persons with information on race and ethnicity, 6,879 (32%) identified as Black, 6,628 (31%) as Hispanic, and 6,330 (30%) as White. From May 17–July 17 to July 18–October 6, the percentage of cases among Black persons increased 67%, from 21% to 35%, whereas the percentage of cases among Hispanic persons decreased 6%, from 33% to 31%. Among White persons, the percentage of cases decreased 28%, from 39% to 28% (Supplementary Figure 1; https://stacks.cdc.gov/view/cdc/121988).

**TABLE:** Epidemiologic and demographic characteristics of persons with monkeypox — United States, May 17–October 6, 2022

Characteristic	No. (%),* by date^†^		
	May 17–Jul 17 n = 3,576	Jul 18–Oct 6 n = 22,807	Total N = 26,384
**Age group, yrs**			
≤12	3 (0.1)	32 (0.1)	**35 (0.1)**
13–17	3 (0.1)	71 (0.3)	**74 (0.3)**
18–29	707 (19.8)	6,016 (27.2)	**6,724 (26.2)**
30–39	1,594 (44.8)	9,084 (41.1)	**10,678 (41.6)**
40–49	826 (23.2)	4,481 (20.3)	**5,307 (20.7)**
50–64	402 (11.3)	2,285 (10.3)	**2,687 (10.5)**
≥65	27 (0.8)	146 (0.7)	**173 (0.7)**
Missing	14	692	**706**
**Race and ethnicity**			
American Indian or Alaska Native, NH	8 (0.2)	83 (0.5)	**91 (0.4)**
Asian, NH	140 (4.2)	464 (2.6)	**604 (2.8)**
Black, NH	699 (21.1)	6,179 (34.5)	**6,879 (32.4)**
Hispanic or Latino	1,096 (33.1)	5,532 (30.9)	**6,628 (31.2)**
Native Hawaiian or other Pacific Islander, NH	7 (0.2)	48 (0.3)	**55 (0.3)**
White, NH	1,281 (38.7)	5,049 (28.2)	**6,330 (29.8)**
Multiple races, NH	6 (0.2)	127 (0.7)	**133 (0.6)**
Other race, NH	71 (2.1)	420 (2.3)	**491 (2.3)**
Missing	268	4,905	**5,173**
**Gender identity**			
Men	3,417 (97.0)	20,326 (94.6)	**23,744 (95.0)**
Cisgender	3,404 (96.6)	20,278 (94.4)	**23,683 (94.7)**
Transgender	13 (0.4)	48 (0.2)	**61 (0.2)**
Women	87 (2.5)	999 (4.7)	**1,086 (4.3)**
Cisgender	44 (1.2)	853 (4.0)	**897 (3.6)**
Transgender	43 (1.2)	146 (0.7)	**189 (0.8)**
Another gender identity	20 (0.6)	151 (0.7)	**171 (0.7)**
Missing	32	536	**568**
**Gender and recent sexual or close intimate contact^§^**			
Cisgender men			
Reported recent MMSC	2,415 (80.4)	8,025 (67.1)	**10,440 (69.8)**
No reported recent MMSC^¶^	40 (1.3)	550 (4.6)	**590 (3.9)**
Reported recent sexual or close intimate contact; partner gender unknown	138 (4.6)	338 (2.8)	**476 (3.2)**
No reported recent sexual or close intimate contact	314 (10.5)	2,177 (18.2)	**2,491 (16.7)**
Transgender men			
Recent MMSC	9 (0.3)	30 (0.3)	**39 (0.3)**
No recent MMSC^¶^	1 (0.0)	3 (0.0)	**4 (0.0)**
Recent sexual or close intimate contact; partner gender unknown	2 (0.1)	1 (0.0)	**3 (0.0)**
No recent sexual or close intimate contact	0 (—)	8 (0.1)	**8 (0.1)**
Cisgender women			
Reported recent sexual or close intimate contact	25 (0.8)	409 (3.4)	**434 (2.9)**
No reported recent sexual or close intimate contact	8 (0.3)	187 (1.6)	**195 (1.3)**
Transgender women			
Reported recent sexual or close intimate contact	31 (1.0)	98 (0.8)	**129 (0.9)**
No reported recent sexual or close intimate contact	3 (0.1)	25 (0.2)	**28 (0.2)**
Another gender identity			
Reported recent sexual or close intimate contact	17 (0.6)	87 (0.7)	**104 (0.7)**
No reported recent sexual or close intimate contact	1 (0.0)	16 (0.1)	**17 (0.1)**
Missing**			
Missing, both	26	471	**497**
Missing, sex and gender	6	65	**71**
Missing, recent sexual or close intimate contact	520	9,522	**10,043**
**No. of sexual or close intimate partners within 21 days before symptom onset** ^§^			
Cisgender men			
One partner	526 (36.3)	2,837 (54.9)	**3,363 (50.8)**
More than one partner	925 (63.7)	2,326 (45.1)	**3,251 (49.2)**
Transgender men			
One partner	4 (80.0)	15 (65.2)	**19 (67.9)**
More than one partner	1 (20.0)	8 (34.8)	**9 (32.1)**
Cisgender women			
One partner	15 (78.9)	224 (72.5)	**239 (72.9)**
More than one partner	4 (21.1)	85 (27.5)	**89 (27.1)**
Transgender women			
One partner	8 (66.7)	35 (59.3)	**43 (60.6)**
More than one partner	4 (33.3)	24 (40.7)	**28 (39.4)**
Another gender identity			
One partner	4 (28.6)	20 (40.8)	**24 (38.1)**
More than one partner	10 (71.4)	29 (59.2)	**39 (61.9)**
Missing/Unknown	2,684	9,591	**12,275**
**Signs or symptoms** ^††^			
Rash	2,783 (99.6)	11,088 (97.8)	**13,871 (98.1)**
Fever	1,367 (72.9)	5,224 (65.7)	**6,591 (67.1)**
Malaise	1,067 (67.6)	4,600 (64.7)	**5,667 (65.2)**
Chills	1,118 (67.6)	4,464 (61.3)	**5,582 (62.4)**
Enlarged lymph nodes	1,061 (63.8)	3,916 (55.8)	**4,977 (57.4)**
Headache	869 (61.4)	4,043 (57.1)	**4,912 (57.8)**
Myalgia	981 (63.8)	3,822 (54.9)	**4,803 (56.5)**
Pruritis	723 (61.5)	3,389 (59.1)	**4,112 (59.5)**
Rectal pain	652 (48.7)	2,597 (39.6)	**3,249 (41.1)**
Rectal bleeding	274 (35.8)	1,289 (21.4)	**1,563 (23.1)**
Tenesmus	253 (24.5)	933 (19.5)	**1,186 (20.4)**
Pus or blood in stools	249 (24.3)	866 (18.1)	**1,115 (19.1)**
Vomiting or nausea	181 (27.6)	853 (17.3)	**1,034 (18.5)**
Abdominal pain	181 (16.3)	847 (14.8)	**1,028 (15.0)**
Proctitis	56 (14.7)	608 (15.7)	**664 (15.6)**
Conjunctivitis	80 (7.7)	195 (4.3)	**275 (4.9)**
**Rash location** ^§§^			
Face	1,219 (43.8)	4,259 (38.4)	**5,478 (39.5)**
Genitals	1,005 (36.1)	4,001 (36.1)	**5,006 (36.1)**
Arms	850 (30.5)	3,713 (33.5)	**4,563 (32.9)**
Trunk	900 (32.3)	3,497 (31.5)	**4,397 (31.7)**
Legs	738 (26.5)	3,277 (29.6)	**4,015 (28.9)**
Perianal	773 (27.8)	2,611 (23.5)	**3,384 (24.4)**
Head	592 (21.3)	2,350 (21.2)	**2,942 (21.2)**
Hands	473 (17.0)	1,921 (17.3)	**2,394 (17.3)**
Neck	287 (10.3)	1,140 (10.3)	**1,427 (10.3)**
Feet	251 (9.0)	1,175 (10.6)	**1,426 (10.3)**
Mouth, lips, or oral mucosa	246 (8.8)	1,115 (10.1)	**1,361 (9.8)**
Other	1,047 (37.6)	3,094 (27.9)	**4,141 (29.9)**
Missing/Unknown location	616 (22.1)	2,658 (24.0)	**3,274 (23.6)**
**HIV infection status** ^¶¶^			
Yes	494 (67.7)	2,382 (55.6)	**2,876 (57.3)**
No	236 (32.3)	1,905 (44.4)	**2,141 (42.7)**
Missing	2,826	17,725	**20,552**

**Abbreviations:** MMSC = male-to-male sexual contact; NH = non-Hispanic or Latino.

* Percentages calculated using nonmissing data.

^†^ July 18 was the day that the last of five commercial labs joined online reporting, expanding *Monkeypox virus* testing capacity to 80,000 per week.

^§^ Includes cases in persons aged ≥18 years with information on gender identity and recent sexual or close intimate contact. Recent sexual or close intimate contact is defined as engaging in any sex (e.g., vaginal, oral, or anal) or close intimate contact (e.g., cuddling, kissing, touching partner's genitals or anus, or sharing sex toys) during the 21 days before symptom onset.

^¶^ Includes men who have had recent sexual or close intimate contact with a cisgender woman, transgender woman, or someone with another gender identity.

** Includes men who have had recent sexual or close intimate contact but are missing information on the gender of their partner.

^††^ Information on signs or symptoms was missing for the following numbers of cases: presence of a rash (12,251), fever (16,560), malaise (17,693), chills (17,442), enlarged lymph nodes (17,706), headache (17,887), pruritis (19,470), myalgia (17,884), rectal pain (18,488), rectal bleeding (19,604), tenesmus (20,570), vomiting or nausea (20,787), pus in stool (20,561), abdominal pain (19,533), proctitis (22,126), and conjunctivitis (20,828).

^§§^ Rash locations are not mutually exclusive.

^¶¶^ Includes cases in persons aged ≥18 years.

**FIGURE 2:**
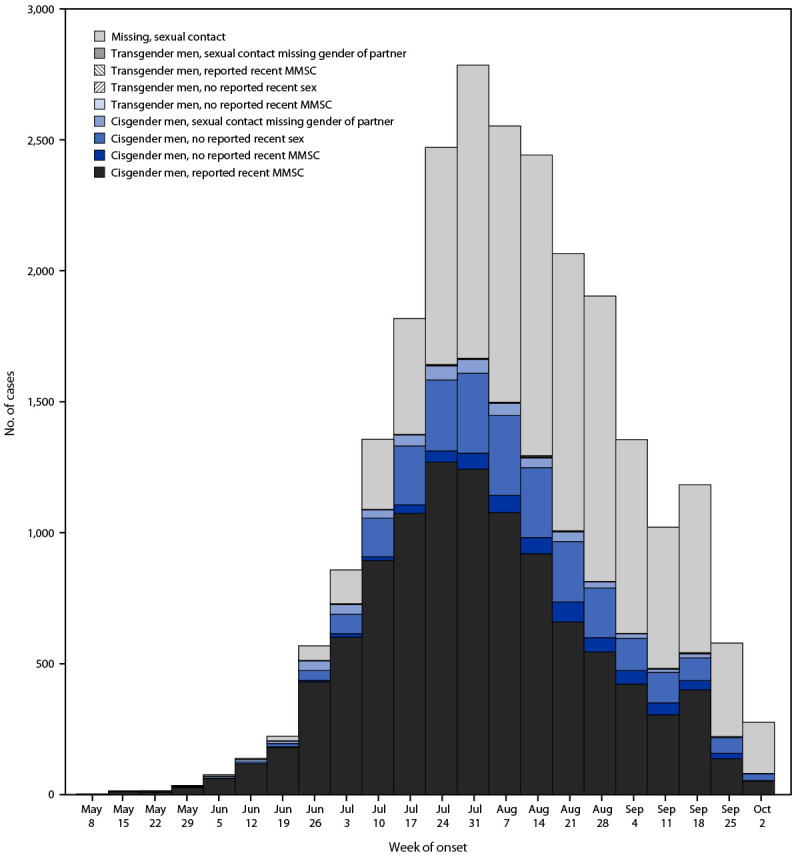
Monkeypox cases* in adult men aged ≥18 years, by week of onset, gender identity, and reported recent sexual contact history — United States, May 17–October 6, 2022 **Abbreviation:** MMSC = male-to-male sexual contact. * Excludes cases in persons aged <18 years and one case with missing information needed to calculate report date. Recent sexual contact is defined as engaging in any sex (e.g., vaginal, oral, or anal) or close intimate contact (e.g., cuddling, kissing, touching partner's genitals or anus, or sharing sex toys) during the 21 days before symptom onset.

Among 25,001 (98%) of 25,569 cases in persons with known age who were aged ≥18 years and for whom information on gender was available, 23,683 (95%) occurred in cisgender men. Cisgender men accounted for up to 99% of cases during the week of June 5; this percentage declined to 80% by the week of October 2 (Supplementary Figure 2; https://stacks.cdc.gov/view/cdc/121989). Among 13,997 cisgender men with information reported on recent sexual or close intimate contact, 10,440 (75%) reported MMSC during the 21 days preceding symptom onset. The percentage of cases in cisgender men who reported recent MMSC decreased over time, from 80% during May 17–July 17 to 67% during July 18–October 6 (Figure 2).

Information on rash was reported by 14,133 (54%) persons with monkeypox, among whom 13,871 (98%) experienced a rash, most frequently on the face (5,478; 40%), genitals (5,006; 36%), and arms (4,563; 33%). Among 5,017 (20%) cases in persons aged ≥18 years with reported information on HIV infection status, 2,876 (57%) had HIV infection. Information on hospitalization was reported for 11,204 (43%) persons with monkeypox, 1,870 (17%) of whom were hospitalized. Six deaths were reported. Information on smallpox or monkeypox vaccination status was provided by 9,197 (35%) persons; 2,882 (31%) had received a vaccine, among whom 1,676 (58%) provided the vaccination date. Among those, 1,670 (99.6%) were vaccinated during the 2022 outbreak (i.e., after May 17). Among those vaccinated during the outbreak with race and ethnicity data available (1,626; 97%), 38% were White, 32% were Black, and 23% were Hispanic. Date of vaccination and date of symptom onset was available for 1,317 (5%) patients, 625 (48%) of whom were vaccinated after symptom onset and 692 (52%) before symptoms began.

## Public Health Response

Within 1 week of announcement of the first suspected case of monkeypox in a U.S. resident by the Massachusetts Department of Public Health in mid-May, CDC launched an emergency response for monkeypox (Figure 1). In addition to enhanced surveillance, the response focused on three strategies: 1) expansion of laboratory testing, 2) use of medical countermeasures for postexposure prophylaxis and treatment, and 3) information dissemination to providers and the public with focus on persons at highest risk within groups most affected by the monkeypox outbreak.

**Laboratory and diagnostic support.** As a result of smallpox preparedness efforts, an FDA-approved polymerase chain reaction laboratory test capable of detecting nonvariola orthopoxviruses was prepositioned through the Laboratory and Response Network (LRN) before the monkeypox outbreak began. In May 2022, 69 LRN laboratories had capacity to test approximately 6,000 specimens per week, and a positive orthopoxvirus test result was sufficient for clinicians and public health departments to initiate patient isolation and contact tracing. On June 10, CDC received FDA clearance to run the same test with a higher throughput extraction platform. To further increase national testing capacity, CDC provided technical assistance to expand testing to five nationwide commercial laboratory companies who began testing during July 2022, increasing the total U.S. testing capacity more than twelvefold, to 80,000 specimens per week (Figure 1). Since the start of the outbreak, testing volume has averaged 9% of capacity in any given week, ranging from 0.2% during the week May 21 to 23% the week of August 20. As of October 8, 2022, a total of 121,345 specimens had been tested.^§§^

**Medical countermeasures.** Licensed vaccines and therapeutics for smallpox are held in the U.S. Department of Health and Human Services Strategic National Stockpile. The JYNNEOS vaccine was stockpiled for immunization of persons who are immunocompromised in the event of a smallpox outbreak. As such, the vaccine was available to help address the monkeypox outbreak. On June 28, a national vaccination strategy was announced to allocate the limited supply of JYNNEOS vaccine based on population-adjusted incidence and the number of persons with highest risk for disease during the current outbreak.^¶¶^ On August 9, 2022, FDA issued an emergency use authorization for JYNNEOS to allow intradermal administration of a lower volume of vaccine to adults aged ≥18 years,*** increasing the number of available doses by a factor of 3–5. As of October 10, 2022, a total of 931,155 doses had been administered (*3*).

Currently, no FDA-approved products for treatment of monkeypox exist. Tecovirimat (Tpoxx) was approved for treatment of smallpox in children and adults during 2018 under FDA’s animal rule, which permits efficacy findings from well-controlled animal studies to support an FDA approval when doing so is not feasible or ethical to conduct a human efficacy trial,^†††^ and is available under an expanded access protocol held by CDC. Tecovirimat is recommended for patients with, or at risk for, severe monkeypox. To reduce disease incidence and facilitate appropriate use of tecovirimat, CDC and FDA reduced the number of required forms and patient samples required for approval of treatment and gave patients the option to see their doctor virtually. A randomized controlled trial led by the National Institutes of Health and the AIDS Clinical Trials Group is underway to determine effectiveness of tecovirimat for treatment of monkeypox, and patients with monkeypox in geographic areas with study sites for this trial are encouraged to enroll.^§§§^

**Provider and public outreach.** CDC uses a variety of mechanisms to disseminate information to public health partners and health care providers about the monkeypox outbreak. On May 20, 2022, CDC released the first Health Alert Network Health Advisory on the monkeypox outbreak,^¶¶¶^ followed by four subsequent advisories. CDC has released clinical considerations for vaccination, treatment, and pain management,**** as well as patient information on treatment and guidance for persons living with HIV infection, women who are pregnant, and children.^††††^ CDC regularly hosts Clinician Outreach and Communication Activity^§§§§^ calls, during which subject matter experts provide the latest information and considerations for clinicians to help them identify potential cases, request testing, and care for patients with monkeypox. Approximately 10,000 health care professionals attended the first call, and 6,000 attended the second.

CDC’s community engagement strategy focuses on harm reduction messaging for populations disproportionately affected by the monkeypox outbreak. Building on existing partnerships from CDC’s initiative to end the HIV epidemic by 2030, CDC has engaged with advocates, experts, and groups focused on sexual health of MSM, transgender, non-binary, gender-diverse, queer, and persons of other sexual identities to directly reach and hear from populations disproportionately affected by monkeypox. For example, on October 20, 2022, staff members from CDC’s Monkeypox Response cohosted an Instagram Live session to promote the Vaccine Equity Pilot Program to agencies that serve racial and ethnic minority lesbian, gay, bisexual, transgender, queer or questioning, intersex, asexual, and others (LGBTQIA+) youth organizations. CDC developed communication resources to facilitate conversations about safer sex and social gatherings, which aim to reduce stigma and aid in making informed choices when persons are in situations or places where monkeypox is more likely to spread. CDC has also supported pilot programs to ensure that vaccines reach populations that have historically faced health disparities and inequity and might continue to face additional barriers to access.

## Discussion

Before the 2022 outbreak, monkeypox cases were primarily reported in central and western Africa.^¶¶¶¶^ In contrast, 37% of all global cases reported as of October 6, 2022, during the current monkeypox outbreak were in the United States.***** Previous knowledge of monkeypox and sexually transmitted infections, laboratory and diagnostic supports, medical countermeasures, and provider and public outreach have been critical components of CDC’s public health response. The unprecedented nature of this outbreak has required flexibility and ongoing assessment of evidence to develop current prevention and treatment practices.

Monkeypox continues to disproportionately affect gay, bisexual, and other MSM. However, the large proportion of cases with missing data on recent sexual or close intimate contact makes generalization of this report’s findings challenging. Tailored, nonstigmatizing communication^†††††^ about risk, transmission, and prevention (*4*,*5*) remains a priority during this outbreak. CDC partners with communities to reduce stigma in communication, emphasizing use of inclusive language and prevention and treatment strategies to reduce fear and encourage action.

The current outbreak also continues to disproportionately affect persons with HIV infection (*6*). In light of data suggesting a higher risk for severe monkeypox disease among persons with advanced and uncontrolled HIV infection (*6*,*7*), it is recommended that persons with suspected monkeypox be offered HIV testing when they initially seek care. Clinicians should consult CDC’s interim guidance on prevention and treatment of monkeypox to potentially reduce severe disease among patients with HIV infection (*7*). Although six deaths have been reported in the United States and other deaths are currently under investigation,^§§§§§^ the case fatality rate for monkeypox during this outbreak remains low.^¶¶¶¶¶^

Multiple strategies exist that can help prevent and reduce transmission of monkeypox. Two vaccines, JYNNEOS and ACAM2000, can be used to prevent infection with monkeypox. During this outbreak, JYNNEOS has been used nearly exclusively because of its favorable adverse event profile overall as well as its favorable outcomes in persons who might be immunosuppressed or have other risk factors for severe monkeypox disease such as atopic dermatitis, heart disease, or a serious vaccine component allergy (*8*). JYNNEOS has not been previously well studied in persons exposed to, or at risk for, monkeypox, although early evidence suggests that vaccination provides some protection against infection (*9*).

The findings in this report are subject to at least three limitations. First, a substantial amount of case data is missing, which might reduce representativeness of findings reported here. Only 59% of adult cases had information on both gender identity and reported recent sexual or close intimate contact. Second, the percentage of missing data is higher in certain jurisdictions; thus, these findings might overrepresent cases from states with more complete data. Finally, mild cases might be underrepresented in this analysis because affected persons might not have sought testing or treatment.

CDC continues to evaluate new evidence and intervention strategies for monkeypox prevention and control based on available data. Public health prevention efforts should emphasize vaccination for persons at high risk for *Monkeypox virus* exposure and prioritize populations most affected by the current outbreak, including MSM, Black and Hispanic persons, and persons who are immunocompromised. Efforts should also focus on reducing stigma when communicating about monkeypox transmission and ensuring equitable access to testing, vaccination, and treatment options to reduce health disparities.

**Summary**

**What is already known about this topic?**

An earlier analysis of 2,891 U.S. monkeypox cases found that up to 99% occurred in men, 94% of whom reported male-to-male sexual contact.

**What is added by this report?**

CDC’s emergency response focused on surveillance, laboratory testing, medical countermeasures, and education. A total of 26,384 U.S. monkeypox cases were reported during May 17–October 6, 2022. Among 59% of persons with data on gender and recent sexual or close intimate contact, 70% reported recent male-to-male sexual contact. Black and Hispanic persons continue to be disproportionately affected.

**What are the implications for public health practice?**

Public health monkeypox prevention efforts, including vaccination, should continue to prioritize gay, bisexual, and other men who have sex with men, Black and Hispanic persons, and persons who are immunocompromised.

## Footnotes

*
https://www.cdc.gov/poxvirus/monkeypox/clinicians/case-definition.html

† Persons whose reported sex differed from their gender or who reported being transgender were classified as transgender. Among cases with reported information on sex but not gender, sex was used to categorize persons as cisgender women or men. Among cases with reported information on gender but not sex, gender was used to categorize persons as cisgender women or men. Among 25,001 adults with information on sex or gender, 3,589 (14%) only had information on sex, and 6,142 (25%) only had information on gender.

§ Sexual or close intimate contact is defined as engaging in any sex (e.g., vaginal, oral, or anal) or close intimate contact (e.g., cuddling, kissing, touching partner's genitals or anus, or sharing sex toys) during the 21 days before symptom onset.

¶ Persons who indicated Hispanic ethnicity, regardless of race, were categorized as Hispanic or Latino. Persons missing data on ethnicity were assumed to be non-Hispanic.

**
https://www.cdc.gov/poxvirus/monkeypox/pdf/scrf-short-form.pdf

†† 45 C.F.R. part 46, 21 C.F.R. part 56; 42 U.S.C. Sect. 241(d); 5 U.S.C. Sect. 552a; 44 U.S.C. Sect. 3501 et seq.

§§
https://www.cdc.gov/poxvirus/monkeypox/response/2022/2022-lab-test.html

¶¶ As estimated by the number of MSM living with HIV infection or persons within a jurisdiction for whom HIV preexposure prophylaxis was recommended.

***
https://www.fda.gov/media/160774/download

†††
https://www.fda.gov/news-events/press-announcements/fda-approves-first-drug-indication-treatment-smallpox

§§§
https://www.stomptpoxx.org/main

¶¶¶
https://emergency.cdc.gov/han/2022/han00466.asp

****
https://emergency.cdc.gov/han/2022/han00471.asp

††††
https://emergency.cdc.gov/han/2022/han00475.asp

§§§§
https://emergency.cdc.gov/coca

¶¶¶¶
https://www.cdc.gov/poxvirus/monkeypox/about/index.html

*****
https://www.cdc.gov/poxvirus/monkeypox/response/2022/world-map.html

†††††
https://www.cdc.gov/poxvirus/monkeypox/resources/reducing-stigma.html

§§§§§
https://www.cdc.gov/poxvirus/monkeypox/response/2022/index.html

¶¶¶¶¶
https://www.cdc.gov/poxvirus/monkeypox/about/faq.html
