# *Erratum:* Vol. 71, No. 45

**DOI:** 10.15585/mmwr.mm7203a7

**Published:** 2023-01-20

In the report “Epidemiologic Features of the Monkeypox Outbreak and the Public Health Response — United States, May 17–October 6, 2022,” on page 1450, the last sentence of the second full paragraph should have read, “Date of vaccination and date of symptom onset was available for **1,563** (**6%**) patients, **610** (**39%**) of whom were vaccinated after symptom onset and **953** (**61%**) before symptoms began.”

